# Physiological Responses During Multiplay Exergaming in Young Adult Males are Game-Dependent

**DOI:** 10.1515/hukin-2015-0054

**Published:** 2015-07-10

**Authors:** Stephen McGuire, Mark ET Willems

**Affiliations:** 1 University of Chichester, Department of Sport & Exercise Sciences, College Lane, Chichester, PO19 6PE, United Kingdom.

**Keywords:** health promotion, active video games, energy expenditure, competition, intensity

## Abstract

Regular moderate-intensity exercise provides health benefits. The aim of this study was to examine whether the selected exercise intensity and physiological responses during exergaming in a single and multiplayer mode in the same physical space were game-dependent. Ten males (mean ±SD, age: 23 ±5 years, body mass: 84.2 ±15.6 kg, body height: 180 ±7 cm, body mass index: 26.0 ±4.0 kg·m^−2^) played the games Kinect football, boxing and track & field (3 × ∼10 min, ∼ 2 min rest periods) in similar time sequence in two sessions. Physiological responses were measured with the portable Cosmed K4b^2^ pulmonary gas exchange system. Single play demands were used to match with a competitive opponent in a multiplay mode. A within-subjects crossover design was used with one-way ANOVA and a post-hoc t-test for analysis (p<0.05). Minute ventilation, oxygen uptake and the heart rate were at least 18% higher during a multiplayer mode for Kinect football and boxing but not for track & field. Energy expenditure was 21% higher during multiplay football. Single play track & field had higher metabolic equivalent than single play football (5.7 ±1.6, range: 3.2–8.6 vs 4.1 ±1.0, range: 3.0–6.1, p<0.05). Exergaming in a multiplayer mode can provide higher physiological demands but the effects are game-dependent. It seems that exergaming with low intensity in a multiplayer mode may provide a greater physical challenge for participants than in a single play mode but may not consistently provide sufficient intensity to acquire health benefits when played regularly as part of a programme to promote and maintain health in young adults.

## Introduction

Regular exercise reduces the risk for the onset and progression of a range of diseases such as cardiovascular disease (CVD), coronary artery disease (CAD), type II diabetes mellitus, osteoporosis and some cancers, specifically colon and breast cancers (Eyre et al., 2004; Garber et al., 2011; Thompson et al., 2003). Recommendations for exercise to provide health benefits are for at least 30 min of moderate intensity exercise five days a week (Garber et al., 2011; Thompson et al., 2003; Haskell et al., 2007). Exercise intensity can be quantified by the metabolic equivalent (MET); MET-values are calculated as multiples of average resting oxygen uptake of 3.5 ml·min^−1^·kg^−1^ (Byrne et al., 2005). In young adults, METs between 4.8 and 7.1 represent exercise of moderate intensity when compared to relative *V*O_2max_ (Garber et al., 2011).

A common given reason for not exercising, especially in already overweight individuals, is a feeling of social anxiety regarding their own body weight, which restricts them from adopting many traditional forms of exercise (Crawford and Eklund, 1994). Further, there are also a large number of people who develop an apathetic nature towards exercise simply from a lack of enjoyment (Hagberg et al., 2008). Thus, alternative methods of increasing exercise participation are required. One such alternative combines new-age video games with exercise, known as an exergame. An exergame is an interactive video game that requires participants to exercise or be physically active in order to play the game. In western societies, despite a causal link being disputed, there is a relationship between obesity and use of electronic media (Robinson, 1999; Vandewater et al., 2004). Indeed, Wright et al. (2001) found that in American children, sleep aside, the use of electronic media is their single largest activity. Thus, there is a clear opportunity for promoting exercise by incorporating exergaming into a seemingly already habitual behaviour. Exergaming has been shown to increase enjoyment and self-efficacy (Yim and Graham, 2007) and it allows people to exercise within their own home, thus potentially alleviating social anxiety and increasing participation.

It is not surprising that many issues of health promoting video games have not been addressed considering the availability of the different game systems with unique playing style requirements. Video exergaming with game consoles such as Microsoft’s Xbox Kinect™ and the Nintendo Wii™ are popular and with high ownership among the general population. The Kinect was chosen for this study as it does not require a controller, thus forcing energy contributions from the entire body (O’Donovan et al., 2012).

Interestingly, multiplay exergaming has rarely been studied despite a key attraction of exergames being the ‘party’ style play they offer. In traditional, non-active video games, competition has been found to be a key influence on a person’s enjoyment (Vorderer et al., 2003), and research has shown enjoyment to be a large influence on exercise adherence (Dishman et al., 2005; Scanlan et al., 1993). In addition, a multiplay mode may increase the physiological responses of the sympathetic nervous system, including a rise in the heart rate and cardiac output (Cooke et al., 2011; Harrison et al., 2001; Matsumura et al., 2011), thus increasing energy expenditure. O’Donovan et al. (2012) reported competitive Microsoft’s Xbox Kinect™ exergaming to result in higher energy expenditure for Reflex Ridge. Peng and Crouse (2013) reported differences in psychological responses comparing multiplayer modes in the same or separate physical spaces. To the best of our knowledge, the effects of the type of game on the physiological and metabolic responses in a multiplayer mode in the same physical space have not been examined.

Thus, the aim of the present study was to investigate whether physiological and metabolic responses on the Microsoft’s Xbox Kinect™ are game-dependent by comparing three activities of the Xbox Kinect Sports game (football, boxing and track and field) in single-player and multiplayer modes. It was hypothesized that multiplay exergaming in the same physical space would enhance similar physiological responses and would not be game-dependent.

## Material and Methods

### Participants

Ten physically active healthy men participated in the study (see Table 1 for subject characteristics). The participants had no history of seizures or epilepsy, were informed of the product safety information, and provided written informed consent. All procedures were approved by the Research Ethics committee of the University of Chichester. The subjects abstained from caffeine and alcohol for twelve hours and complete fast for three hours before testing (O’Donovan et al., 2012). They did not have any previous experience with the Kinect and the Kinect Sports game. The participants were tested in single play and, one week later, against another participant in the same physical space in a multiplayer mode. Body height and mass were measured to the nearest 0.1 cm and 0.1 kg, respectively, with participants lightly clothed but shoeless. The body mass index (BMI) was calculated as body mass (kg) divided by height squared (m^2^). All participants were instructed on use of the Xbox’s Kinect system and the Kinect Sports game. They were then familiarised with each of the three games played by following the in-game instructions; this approach ensured a standardized level of competency among the participants.

Testing was conducted using a Microsoft Xbox and Xbox Kinect accessory, which utilises a motion detection camera to track player movements without the use of a physical controller. The participants played through a series of three activities (i.e. football, boxing and track and field) from the Kinect Sports game, with game time totalling 30 minutes of physical activity, in accordance with physical activity, public health and exercise recommendations (Garber et al., 2011; Haskell et al., 2007), excluding periods of inactivity (between game loading time). Football and boxing are played with primarily lower and upper body movements, respectively, whereas track and field is a sequence of upper and lower body movements depending on the event. Gaming time allowed, in the following order, two matches of football, at least two bouts of boxing and one track and field circuit. Difficulties were selected to be challenging, based on the familiarisation session, but the hardest difficulty was deemed too challenging for novice game users and not used to avoid demotivating participants. The activities were chosen as the most physically demanding of the activities available and the order was selected as a progression in intensity. Randomization was not done to avoid potential effect of the game with highest intensity on the physiological and metabolic responses of subsequent lower intensity games.

### Football

In the first game, the participants, when in possession of the football, needed to make a kicking movement in the direction for passing or shooting (depending on their position on the pitch). As a defender, the participants reacted to the direction of the opponents pass and sidestepped into the path of the ball, as indicated by an on-screen arrow. As a goalkeeper, they must use any part of their body to block the shot of their opponent. The degree of difficulty in this game adjusts the speed of the computer and the difficulty of scoring past it, thus forcing the participant to react quicker by increasing the speed of their side steps and kicking, effectively forcing elevated activity levels to cope with the computers increased speed. Two games were played and game difficulty either increased or remained the same if they won or lost, respectively. During multiplayer, the participants shared half of the screen split vertically and reacted, in the same manner as playing the computer, to the movements of their competitor.

### Boxing

For boxing, the participants were required to punch and block the computer opponent, punching high and low to generate successful combinations; power shots could also be achieved through punching with a strong rotation of the shoulder. As in real boxing, one could win either by knocking out their opponent or on a points decision, and lose in the same manner. A minimum of two games were required to meet 10 minutes of activity, the game was restarted if resolved early by a knock out, either at the next level of difficulty or the same level of difficulty if they won or lost, respectively. The level of difficulty in this game dictated the speed and defensive capabilities of the computer, forcing the participants to punch quicker and for longer, as well as to react quicker to counter punches from the computer. During multiplayer, the participants had the same view but again on half the screen, with the opponent’s actions controlled by their human competitor. If one participant was knocked out the game was restarted, until ten minutes of play were complete.

### Track and Field

Track and field consisted of 6 consecutive events. For sprint, speed was dictated by the speed and height the participant could raise their knees whilst running in place. For javelin, the distance thrown was a product of approach velocity and the point of release, both in relation to distance from the foul line and the angle of the arm when releasing the javelin. For the long jump, the approach velocity and timing (of the jump) determined the performance. For discus, a rapid throwing movement was required for the best distances. For hurdles, the same speed rules applied as the sprint event, but the participants also had to incorporate a jump to avoid the hurdles, and an accompanying reduction in speed. For a rapid runner, participants were running in place between work and rest zones. A timer continuously counted down and the participants earned more time by reaching the next rest zone. As running progressed, the rest zones got further apart, forcing the subjects to run faster.

In all games, with the exception of the ‘rapid runner’ in which the participants dictated the intensity through personal effort, the difficulty affected the scores of the computer forcing the subjects to work harder to beat it. As this activity averaged 10 minutes for all the events, only one game was played at the third difficulty level. Multiplayer gaming in this event again utilised split-screen for races, but solo events (javelin, long jump, discus and rapid runner) had to be played alone with the participants taking turns.

**Figure 1:**
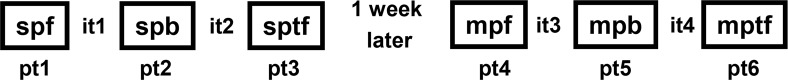
Order of play for single and multiplayer settings, playing time and inactivity time. spf, single player football; spb, single player boxing; sptf, single player track and field; mpf, multiplayer football; mpb, multiplayer boxing; mptf, multiplayer track and field; pt1, playing time spf (10:13±00:39 min); pt2, playing time boxing (10:40±02:17 min); pt3, playing time track and field (10:16±01:15 min); pt4, playing time multiplayer football (11:04±00:43 min); pt5, playing time multiplayer boxing (09:35±01:50 min); pt6, playing time multiplayer track and field (13:21±02:19 min), it1, inactivity time 02:30±01:09 min; it2, inactivity time 02:28±00:30 min; it3, inactivity time (02:03±00:23 min); it4, inactivity time (02:24±00:12). The increased time of track and field in the multiplayer condition gameplay was due to some games having to be played in turns for this activity.

Following a warm-up, the participants completed the three games in order (football, boxing, track and field) without encouragement. The game had unavoidable breaks between games (i.e. loading time), but activities were completed in at least 10 minute blocks. Playing times are provided in Figure 1.

### Multiplayer

In the multiplayer mode, the participants were matched against a participant of similar ability and played through the same games in the same order. No encouragement was given to either participant, with motivation solely coming from a desire to win.

### Physiological and metabolic responses

Physiological and metabolic responses were measured using a Cosmed K4b^2^ (Cosmed Srl; Rome, Italy) and HR monitor (Polar heart rate monitors, Polar Electro Ltd, Warwick, UK). The K4b^2^ is a portable, battery operated, metabolic measurement system using a breath by breath system. Attached by a harness, the K4b^2^ with a battery weigh 550 g each (McLaughlin et al., 2001) and was non-restrictive for the game movements. Expired air was analysed via a turbine attached to a close fitting face mask covering the participant’s mouth and nose, preventing any exhaled air escaping. The Cosmed was calibrated before each testing session, following manufacturer guidelines, to gases of known concentrations (oxygen, O_2_: 15.15%, carbon dioxide, CO_2_: 5.03%), room air, a delay calibration of expired oxygen and carbon dioxide and a respiratory volume calibration using a three litre syringe. Data from the K4b^2^ was downloaded to Microsoft Excel to allow calculation of minute ventilation (*V̇_E_*), oxygen uptake (*V̇O*_2_), the heart rate (HR), energy expenditure (EE) and the respiratory exchange ratio (RER). Energy expenditure was expressed in kcal. Exercise intensity was expressed in MET. One metabolic equivalent equals a metabolic rate of 3.5 ml O_2_·kg^−1^·min^−1^ (Jetté et al., 1990). The Cosmed had been validated at both low and moderate intensities (Schrack et al., 2010). In addition, McLaughlin et al. (2001) reported negligible differences in oxygen uptake at all intensities (9.6% difference from Douglas bag measures at 50 W and less than 3% at 200 W). Furthermore, the K4b^2^ had been reported as a reliable tool at low intensities (Schrack et al., 2010), with Duffield et al. (2004) concluding it had satisfactory test-retest results with good repeatability of minute ventilation, oxygen uptake and carbon dioxide removal.

### Data analysis

SPSS (SPSS statistics 20, IBM Corp., Armonk, NY, USA) was used for statistical analysis. Physiological responses were analysed with between game reloads (i.e. the reload time between game one and two of a particular activity and with track and field waiting time as single and multiplay were not played in real time). Oneway ANOVA with post-hoc t-testing was used to analyse physiological responses and metabolic equivalent values. Power analysis indicated a sample size of 8 to detect an increase of 20% of MET when playing football in single play compared the multiplay mode with a high statistical power (1 – β = 0.80: 0.05 = α level). Interpretation of 0.05 > *p* ≤ 0.1 was according to guidelines by Curran-Everett and Benos (2004). Effect sizes (Cohen’s *d*) were calculated for the multiplay mode and interpreted as small (0.2 to 0.3), moderate (0.31 to 0.79) and large (≥ 0.8). The level of statistical significance was set at p<0.05.

## Results

### Physiological and metabolic responses

Physiological and metabolic responses are shown in Table 2. Minute ventilation, oxygen uptake, heart rate and energy expenditure in the multiplayer football condition were 25% (p=0.002, *d*=0.87), 18% (p=0.009, *d*=0.61), 37% (p=0.002, *d*=1.69) and 21% (p=0.004, *d*=0.75) higher, respectively, compared to single-player football. An increase was also observed in the multiplayer mode boxing for minute ventilation (+32%, p=0.008, *d*=0.68), heart rate (+30%, p=0.021, *d*=1.15), energy expenditure (+27%, p=0.031, *d*=0.62) and respiratory exchange ratio (+0.07 units, p=0.034, *d*=1.37), with a trend for higher oxygen uptake (+25%, p=0.057, *d*=0.66). There were no differences in physiological and metabolic responses between single and multiplayer modes for the track and field activities.

### Metabolic equivalents

Metabolic equivalents are shown in Table 2. The metabolic equivalent of single play football was lower than single play track and field. Within games, the metabolic equivalent of multiplay football was 15% (p=0.019, *d*=0.66) higher than single play with a trend for multiply boxing to be 16% higher (p=0.081, *d*=0.62). Individual responses showed that 8 players in single play football did not reach a MET value higher than 4.8. In addition, of those 8 players, multiplay football did allow 2 players to have a value higher than 4.8. For boxing, 5 players in the single play mode did not reach a MET value higher than 4.8 with 3 of those reaching values higher than 4.8 in the multiplay mode. For track and field, 9 players reached a MET value higher than 4.8 with 2 of those having lower MET values than 4.8 in the multiplay mode.

## Discussion

The aim of the present study was to examine the effect of playing in a single or multiplayer mode in the same physical space in games played with different intensity (i.e. football, boxing and track and field) on metabolic and physiological responses using the Xbox Kinect Sports game. We hypothesised that the physiological and metabolic response would be higher in the multiplayer condition. The primary finding of the present study was that the physiological and metabolic responses could be higher in the multiplayer mode but were game-dependent with no differences for track and field (i.e. the game with the highest metabolic equivalent) between the single and multiplay mode. However, the similarity for the single and multiplay mode for track and field may be due to not playing in real time and waiting time responses were included in the comparison of the play mode. Moderate and large effect sizes occurred for the physiological and metabolic responses that were significantly larger during the multiplay mode for football and boxing. A study by Scheer et al. (2014) showed a similar heart rate for Kinect boxing (i.e. 119 b·min^−1^) for a sample of 10 males and 9 females than for single play boxing in the present study (i.e. 120 b·min^−1^). However, comparison with other exergaming studies is problematic due to differences in game styles, participants and equipment mechanics. The Kinect was shown to provide greater energy expenditure than its nearest market competitor, the Nintendo Wii due to energy contributions from a greater range of body parts (O’Donovan et al., 2012).

Anecdotal observations during single play football indicated a lack of enjoyment or dissatisfaction during single play but not in multiplayer modes. Greater levels of enjoyment have been linked with greater exercise intensity (Dyrlund and Wininger, 2008) and enjoyment has been shown to mediate the effects of competition (Cooke et al., 2011); this is likely responsible for the changes seen for the physiological and metabolic responses in the multiplay mode for football and boxing. Previous research showed boxing on the Nintendo Wii (single player mode) to be a more physical demanding game than tennis and baseball (Willems and Bond, 2009). In the present study, multiplay boxing on the Kinect displayed significant changes in physiological and metabolic responses. This was likely caused by a change in the mechanics of the game in the multiplayer mode. When playing alone, the participants were forced to include blocking actions and play at the pace of the computer in order to be successful. By contrast, in the multiplayer mode all participants opted for an intense, erratic style of all out punching in a desire to win as quickly as possible. This finding demonstrates the potential of competitive exergaming as the level of intensity could not be physically achieved in single-player gameplay without consistently losing; this may cause demotivation. However, this style of boxing in the multiplay mode drastically reduced individual game time and it is unclear whether participants would lose motivation to play for the required time outside of a laboratory setting.

For track and field, there were no differences in physiological and metabolic responses for play in the single and multiplayer mode. We can only speculate that this was partly due to this game being played with a higher intensity. A potential reason for not having a difference between the single and multiplay mode for track and field may be due to the characteristics of this game on the Kinect. The track and field game simply involved throwing actions or running and jumping; unlike the other two games, there was no ‘trick’ to good performance. This assumption is supported by previous research which has shown intensity to be mediated by efficacy (McAuley, 1992; Sallis et al., 1986).

The secondary finding of the present study was that the multiplay mode of low-intensity games may be beneficial for some participants to reach MET values that are in line with recommended activity levels for young adults (4.8–7.1 METs). Our participants were considered recreationally active males, thus it is possible that the low-intensity game of football was not challenging considering their fitness level. In addition, a meta-analysis by Peng et al. (2011) of 18 studies showed that energy expenditure was found to be significantly moderated by player age and the type of game played but no data on Xbox Kinect was available at that time. Given these findings and the apparent lack of Kinect data, future studies should address the physical demands of a mulitplay mode with different games and different populations.

Participants in the present study were only males to avoid a possible Hawthorne effect as males seem to gain a dominant advantage in a competitive environment in mixed gender events (Gneezy et al., 2003). A potential limitation of the study was that our subjects were novel to the Kinect games. However, they were familiarized with the Kinect games, but we cannot exclude that the physiological and metabolic responses were partly due to novelty effects. Another limitation is that the resting oxygen uptake was not individualized for each subject ([Bibr b29-jhk-46-263][Bibr b30-jhk-46-263]).

It is concluded that multiplayer exergaming provides higher physiological and metabolic demands compared to a single play mode but the effect seems to be game-dependent. It seems that low intensity exergaming in a multiplayer mode may provide a greater physical challenge for participants but does not consistently provide sufficient intensity to be able to get health benefits when played regularly as part of a programme to promote and maintain health.

## Figures and Tables

**Table 1:** Subjects characteristics

Age (years)	23 ±5
Body height (cm)	180 ±7
Body mass (kg)	84.2 ±15.6
BMI (kg·m^−2^)	26.0 ±4.0

**Table 2:** Physiological and metabolic responses in 10 minute bouts of Xbox Kinect Sports game activities, played in single-player and multiplayer game modes

	**Football**		**Boxing**		**Track and field**	

	SP	MP	SP	MP	SP	MP
*V̇_E_* (L·min^−1^)	31.9 ±9.0	38.9 ±9.2^*^	40.1 ±14.1	51.7 ±19.4^*^	54.2 ±12.4	55.5 ±10.6
*V̇O*_2_ (ml·kg^−1^·min^−1^)	14.6 ±3.8	16.6 ±2.6^*^	16.3 ±4.5	19.1 ±4.0^#^	19.7 ±4.8	18.8 ±2.6
HR (b·min^−1^)	82 ±30	122 ±15^*^	120 ±28	147 ±18^*^	125 ±21	136 ±15
EE (kcal)	57.5 ±14.7	68.2 ±13.8^*^	67.5 ±21.3	82.9 ±27.8^*^	82.1 ±15.5	79.9 ±11.9
RER	0.86 ±0.08	0.91 ±0.04	0.96 ±0.06	1.03 ±0.04^*^	1.05 ±0.07	1.06 ±0.05
MET	4.1 ±1.0	4.7 ±0.8^*^	4.7 ±1.3	5.5 ±1.1^#^	5.7 ±1.6	5.4 ±0.7

Values are mean ±SD for 10 participants. V̇_E_, minute ventilation; V̇O_2_, oxygen uptake; HR, heart rate, EE, energy expenditure; RER, respiratory exchange ratio; MET, metabolic equivalent.

* Denotes significantly higher in the multiplayer (MP) condition than the activity in a single-player (SP) mode (p<0.05).

# Denotes a trend to be higher in the multiplayer (MP) condition than an activity single-player (SP) mode (0.05 > p ≤ 0.1).
